# Proximal fibular osteotomy in the treatment of medial osteoarthritis of the knee – A narrative review of literature

**DOI:** 10.1186/s43019-019-0016-0

**Published:** 2019-12-18

**Authors:** Saseendar Shanmugasundaram, Srinivas B. S. Kambhampati, Samundeeswari Saseendar

**Affiliations:** 1 Apollo Hospital, Muscat, Sultanate of Oman; 2 Sri Dhaatri Orthopaedic, Maternity and Gynaecology Center, Vijayawada, Andhra Pradesh India; 3 Manakula Vinayagar Medical College and Hospital, Pondicherry, India

**Keywords:** Proximal fibular osteotomy, Medial osteoarthritis, High-fibular osteotomy, Upper partial fibulectomy

## Abstract

Proximal fibular osteotomy has been proposed as a simple and inexpensive alternative to high-tibial osteotomy and unicondylar knee arthroplasty and may be useful for low-income populations that cannot afford expensive treatment methods. However, there is no consensus existing regarding the mechanism by which it acts nor the outcome of this procedure. This study was performed to analyze the available evidence on the benefits of proximal fibular osteotomy and to understand the possible mechanisms in play. There are various mechanisms that are proposed to individually or collectively contribute to the outcomes of this procedure, and include the theory of non-uniform settlement, the too-many cortices theory, slippage phenomenon, the concept of competition of muscles, dynamic fibular distalization theory and ground reaction vector readjustment theory. The mechanisms have been discussed and future directions in research have been proposed. The current literature, which mostly consists of case series, suggests the usefulness of the procedure in decreasing varus deformity as well as improving symptoms in medial osteoarthritis. However, large randomised controlled trials with long-term follow-up are required to establish the benefits of this procedure over other established treatment methods.

## Introduction

Osteoarthritis (OA) of the knee is the commonest form of OA. It occurs commonly in the medial compartment. The reported prevalence of radiographic and symptomatic OA of the knee in elderly persons above 60 years age is estimated to be 37% and 12%, respectively [1] and the lifetime risk of symptomatic knee OA was found to be 44% [2].

Radiological evidence of grade 3–4 OA was seen in up to 34% in women and 31% in men aged above 70 years [3]. The prevalence was 27% in subjects aged less than 70 years going up to 44% in those aged above 80 years [3]. Among recently conducted studies, the prevalence of radiographic OA (≥ grade 2 Kellgren-Lawrence (KL)) was studied in Sweden and found to be 25.4% among 10,000 subjects. Symptomatic OA was found to be 15.4% while frequent knee pain was reported in 25.1% of these subjects [4]. In a study of prevalence in the South Korean population of 2289 subjects, Lee et al. [5] found a prevalence of 13% for knee OA. Age over 65 years, of female sex, with obesity, hypertension, and low frequency of strength exercises were found to be risk factors.

The prevalence in a rural Japanese population of symptomatic knees with radiographic OA (grade 2 and above) was 35.6% and 26.5% among women and men, respectively, with almost all of the knees with radiographic OA exhibiting a varus deformity [6]. Varus deformity is known to be common in primary OA of the knee with a reported incidence as high as 63% reported by Barrett et al. [7] in a population with an average age of 72 years in 2197 weight-bearing radiographs of the knee, and more than 97% in patients with radiographic OA in the rural Japanese population [6].

Established surgical options for the treatment of medial OA include high-tibial osteotomy (HTO), unicompartmental knee arthroplasty (UKA) and total knee arthroplasty (TKA). Proximal fibular osteotomy (PFO) or upper partial fibulectomy is a procedure that has been proposed relatively recently to reduce knee pain in patients with medial OA and is being taken up by an increasing number of orthopedic surgeons. This article attempts to review the existing literature on this procedure in relation to the origin, biomechanics, indications and outcomes of this procedure.

## Methodology of the study

A search in PubMed for “proximal fibular osteotomy” gave 223 results. Of these, 10 studies were directly related to PFO, of which two were in Chinese. A similar search resulted in eight articles from Ovid Medline and 10 articles from Embase, all of which were relevant. A search in Scopus produced 204 results, of which eight were relevant. A search in the Cochrane Library for “proximal fibular osteotomy” gave seven results, only one of which was relevant to PFO. However, the article was not traceable in the archives of the journal.

Articles in languages other than English and articles which combined PFO with other surgical procedures (e.g., HTO, UKA) were excluded. The bibliography of the articles were further searched for relevant studies. After accounting for duplication of articles and overlap of search results, there were 10 articles that assessed the clinical or biomechanical effects of PFO in medial OA of the knee. This also included articles that used the term “upper partial fibulectomy” instead of “proximal fibular osteotomy.” All these articles were published within the past 5 years.

The mechanisms proposed for the development of varus deformity in knee OA and the mechanisms of clinical improvement after PFO were studied. A systematic review was not done as there were few long-term outcome studies of PFO.

## Discussion

### Biomechanics of varus knee osteoarthritis

With increasing grade of OA of the knee, femoral neck-shaft valgus angle decreases and lateral bowing of the femoral shaft increases, reducing the condylar shaft angle and shifting the mechanical axis medially on the femoral side, whereas on the tibial side, tibial plateau compression leads to a steepening of the medial plateau more than bowing of the tibia, especially in the early stages of OA [8]. Bowing of the tibia starts to occur from a moderate grade of OA and progress of medial OA has been found to occur more due to medial tibial compression rather than to bowing of the tibia [8].

The determinants of compressive strength and stiffness of trabecular bone reportedly are the apparent density of trabeculae, trabecular architecture and the strength of the bone material [9]. The primary trabeculae of the epiphysis are oriented perpendicular to the joint surface of the proximal tibia [9].

There is no difference in the age-related changes and mechanical properties of subchondral trabecular bone in the medial and lateral tibial condyles [10]. However, women appear to resorb trabecular bone at a faster rate than men thereby putting them at higher risk than men to collapse of metaphyseal trabecular bone [11, 12].

In the proximal tibia, which is predominantly a cancellous bone, the trabeculae rather than the peripheral cortex share most of the load [9]. Hence, age-related trabecular resorption in the proximal tibia leads to the risk of collapse. The fibula, being a predominantly cortical bone, is not affected by this.

Hvid [9] worked on the loading pattern and strength of the proximal tibia and found that loading and consequently the strength of bone is maximal in the central and anterior sections of the medial tibial plateau, whereas these occur posteriorly in the lateral tibial plateau. He also found the medial side to take more load compared to the lateral side.

There is minimal change in the cortical thickness in the proximal fibula with age but the loss of strength is more significant in the proximal tibia [13]. The loads transmitted by the fibula have been reported to be between 6.5 and 16% of the total load borne by the lower limb [14]. Also, loading of the fibula varied with the position of the ankle and subtalar joints. Maximum fibular loading occurred when the ankle joint was in full dorsiflexion and the subtalar joint was in full eversion [14]. All the above studies indicate that loading through the fibula is relatively well preserved with age and the fibula contributes to supporting the lateral column of the proximal tibia.

### Wear patterns in knee osteoarthritis

It is important to understand the normal wear patterns in OA of the knee as PFO aims to reverse these changes. In osteoarthritic knees with normal alignment and an intact anterior cruciate ligament (ACL), wear is seen commonly in the anteromedial aspect of the medial compartment [15] and posterolateral part of the lateral compartment [16]. The wear patterns correspond with the loading pattern of the proximal tibial articular surface described above by Hvid [9].

In osteoarthritic knees with deficient ACL, the worn area is wider on the medial compartment and involves the posterior aspect of the medial compartment. Varus deformity was significantly greater in the ACL-deficient knee and the severity of the deformity did not alter the wear pattern of the knee [15], irrespective of ACL integrity.

### Origin of proximal fibular osteotomy

The first reported suggestion that fibulectomy results in a decrease in the medial compartmental pressure and an increase in the lateral compartmental pressure was by Yazdi et al. [17] in 2014. They surveyed joint reaction forces across cadaveric knees after fibulectomy for other reasons, e.g., fibular-cuff resection for non-union of the tibia, fibular tumor resection, fibula graft harvest, etc. The authors suggested that performing fibulectomy along with periarticular knee osteotomies can have protective effect by reducing pressure over the knee joint.

The following year, Yang et al. [18] published the results of their retrospective series of PFO surgeries performed since 1996 at the Third Hospital of Hebei Medical University, Hebei, China. Zhang, who is the corresponding author of this study, in a separate interview, attributes the idea of PFO to one of his students from a rural hospital in China [19].

### Rationale behind proximal fibular osteotomy

Many mechanisms appear to interplay after PFO. We discuss each of these below.

#### The concept of non-uniform settlement

The word “settlement” has been borrowed from the field of architecture where the phenomenon of gradual sinking after the construction of a structure is seen [20]. The bone density of the fibula was found to be higher than the medial tibial plateau. With osteoporosis, the support of the fibula by the lateral tibial plateau does not allow the lateral side to “settle” creating a varus deformity. This has been called non-uniform settlement [20]. When the medial side “settles” down, there is side-slipping of the femoral condyle medially during walking and playing sports which aggravates the non-uniform settlement due to excessive loads on the medial side [18].

The rationale behind PFO is that when this support of the fibula is removed, the lateral side “settles” down, loading the proximal tibia evenly and leading to the correction of the deformity in a varus knee, thereby relieving symptoms and reducing the deformity.

**Fig. 1:**
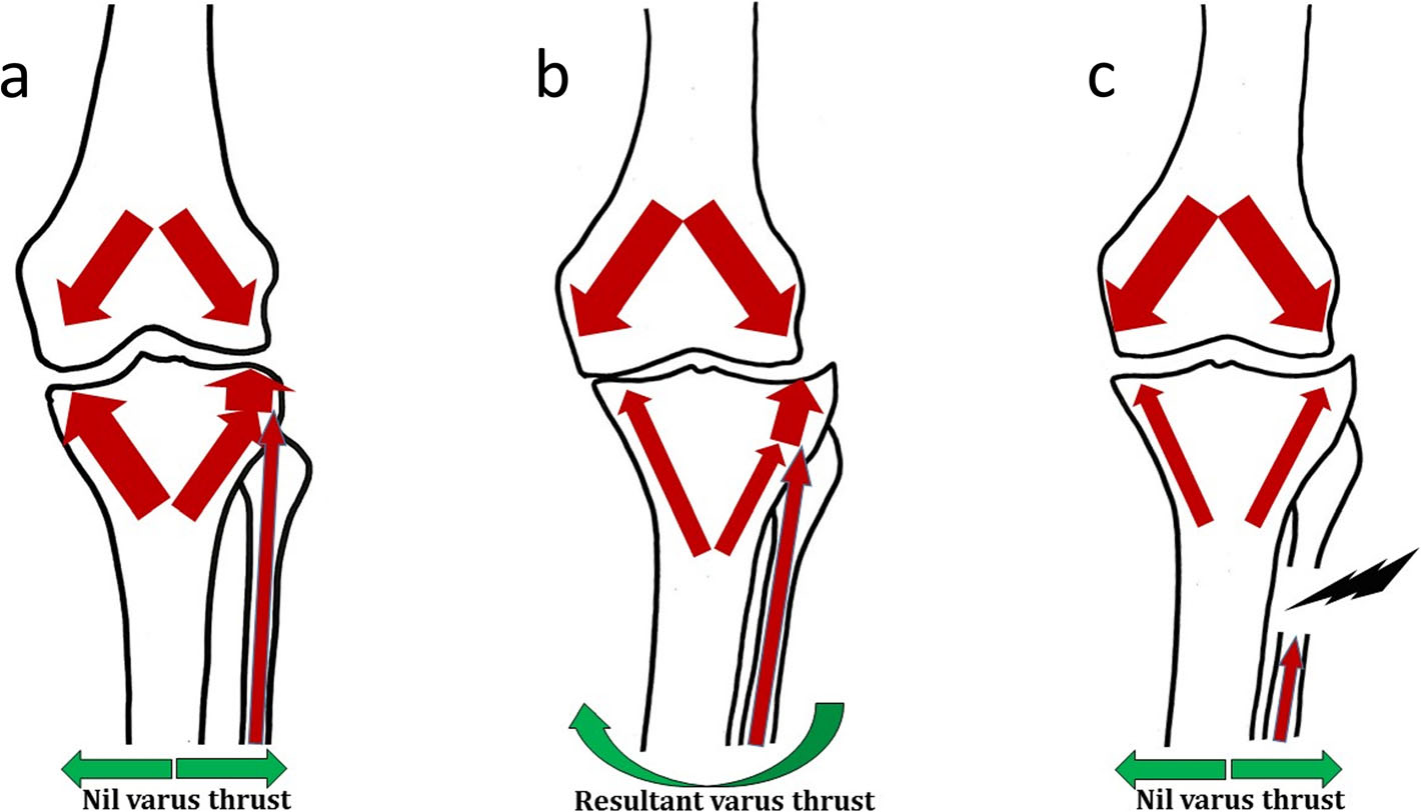
Illustration demonstrating non-uniform settlement on the medial aspect and the “too-many cortices” laterally. Downward arrows depict the load exerted on the knee joint. Upward arrows depict load-bearing by the medial and lateral condyles of the tibia and the fibula. **a** normal knee, **b** varus knee and **c** after proximal fibular osteotomy (PFO) (details in text)

It was found in clinical studies of PFO that varus deformity improved following PFO [18, 19, 24]. When the continuity of the fibula is interrupted, the loads borne by the lateral column of the proximal tibia increases resulting in “settling” on the lateral side [21, 24] (Fig. 1).

Settlement value has been studied by Dong et al. [20] and found to be negatively related to the hip-knee angle (HKA) and positively related to the KL grading of OA of the knee. Settlement value was defined as the distance of the lowest point of the medial condyle of the tibia in an anteroposterior view of the knee, from a perpendicular drawn through the highest point in the lateral tibial condyle [20] to the tibial mechanical axis. It has been found that varus alignment increases the progression of OA [25]. The greater the varus, the greater the chances of progression and hence reducing the varus by PFO decreases the progress of OA.

#### The too-many-cortices theory

Another theory is that the medial condyle is supported by one cortex whereas the lateral condyle is supported by one tibial cortex and two fibular cortices making it difficult to balance loading when the medial side collapses in a varus-deformed knee with an intact fibula [22] (Fig. 1).

Figure 1 depicts the loads borne by the proximal tibia and the fibula in a normal knee (a), a varus knee (b) and after PFO (c). A – normal knee – the medial tibial load-bearing and the cumulative lateral load-bearing (tibial + fibular) are equal to each other (depicted by same-sized arrows), hence there is no varus or valgus thrust; B – an older, arthritic knee – age-related trabecular resorption in the tibia causes a lower load-bearing capability of the tibia (depicted by smaller arrows). The fibula, being a cortical bone, is not affected by trabecular resorption. Hence, the resultant load-bearing is better on the lateral side and weaker on the medial side resulting in trabecular collapse on the medial side, leading to varus deformity; C – post-PFO knee – the trabecular density and, hence, load-bearing capacity is equal between the medial and lateral tibial condyles. The lateral strut effect of the fibula is interrupted by surgery, thereby eliminating the varus thrust.

#### Slippage phenomenon

After the development of varus due to non-uniform settlement, the femur slides to the medial side as is evident in plain radiographs of patients with medial-compartmental OA. This phenomenon is called coronal tibio-femoral subluxation or the slippage phenomenon. This phenomenon further tends to increase the high Knee Adduction Moment (KAM), thereby further enhancing the non-uniform settlement, leading to progression of varus deformity.

#### The concept of competition of muscles

Huang et al. [26] proposed that there was a competition of muscles between biceps femoris and peroneus after high-fibular osteotomy. He found that muscle activity increased in the long head of biceps femoris and decreased in the peroneus longus on the side operated on immediately after high-fibular osteotomy. This explained the immediate improvement in HKA angle from a more varus to a more neutral alignment immediately after high-fibular osteotomy. This finding is significant and explains the immediate pain relief after surgery since correction of the HKA angle from a rectified non-uniform settlement would not be expected to be evident immediately after a fibular resection.

#### Dynamic fibular distalization theory

Qin et al. [27] in their prospective study of 67 PFOs found that significant clinical improvement after surgery was proportional to the amount of distalization of the fibula and the inclination angle of the proximal tibio-fibular joint. The authors conceptualise that after PFO, the proximal fibula was no longer subject to compressive forces of weight transmission from the distal fibula. However, muscles attached to the proximal fibula, such as the soleus and peroneus longus, pulled the fibular head in the distal direction, and the tensile force was simultaneously transmitted from the fibular head to the lateral femoral condyle, thereby narrowing the lateral knee-joint space. This theory was supported by the finding that the greater the distal displacement of the fibular head, the better was the correction of the varus deformity and the more significant the improvement in symptoms.

#### Ground reaction vector readjustment theory

Xie et al. [28] in 2018, attributed immediate symptomatic relief after PFO to biomechanical changes in ground reaction vector (GRV) action mainly at the foot level.

In patients with varus knees, the hindfoot goes into valgus during the stance phase to move the origin of the GRV laterally and, therefore, closer to the center of the knee, reducing the KAM. With an intact fibula, this compensatory valgus is limited. The authors proposed that after PFO, the lateral malleolus migrates proximally, pulling the calcaneus into further valgus through the calcaneofibular ligament. This results in a more laterally directed GRV, thereby relieving pressure on the medial knee and causing instant pain relief. However, the authors have not provided evidence to support their theory.

Guo et al. [29] disproved the above theory in their recent prospective study of 49 patients. There was no proximal migration of the lateral malleolus. They did not note any significant anatomical valgus alignment at the ankle postoperatively, rather ankle valgus improved after PFO.

#### Procedure

Unlike high-tibial osteotomy, the surgical methods for high-fibular osteotomy are more limited. Various approaches have been used to resect a segment of the fibula 6–10 cm below the fibular head.

Huang et al. [26] advocates removing a 1-cm segment of fibula 7–8 cm from the head of the fibula by accessing it through the inter-muscular space between the extensor digitorum longus and peroneus longus/peroneus brevis, under local anesthesia. Others have used an approach between the peroneus and the soleus and removed a 2-cm segment 6–10 cm below the fibular head [20, 30].

The surgical approach should primarily be influenced by course of the common peroneal nerve (CPN) and efforts to minimize damage to the nerve or its branches. Accordingly, the plane of dissection to approach the fibula and the zone of fibula resected are of prime importance.

It has been shown in studies of fibular osteotomies accompanying high-tibial osteotomies that it is safest when the fibula is excised from the lower half. This zone at the junction between the proximal two thirds and the distal third of the fibula had the least incidence of peroneal nerve palsy without compromising the stability of the ankle [31, 32].

When an osteotomy is needed in the proximal half, it should be performed through an incision posterior to the coronal plane to avoid the peroneal nerve and its branches which are in anterior to the coronal plane [32].

It is not presently clear whether resecting the proximal fibula confers any benefits over resecting the fibula from the distal half for patients undergoing isolated fibular osteotomy for medial OA of the knee.

### Outcomes

There was an increase in valgus at the knee of 5° reported following the procedure at 1 year [21]. Huang et al. [21] looked into the gait changes following this procedure in one case and reported an increase in femoral abduction of 5–7° and 5–8° of femoral external rotation at 3 months after the operation. Distal femoral translation also increased by 2–10 mm. Although it is useful in correcting varus to some extent, the varus should be originating from deformity of the proximal tibia rather than the distal femur.

Radiographs at 1 year showed increased valgus of the knee and improvement in the medial joint space in reports by multiple authors [21, 27]. The mechanism by which these changes occur following a PFO is not clear but the theories have been previously discussed.

Wang et al. [24] reported results on 46 patients who had undergone PFO with a follow-up of 12 months. No postoperative complications were found, and the duration of the procedure lasted 32 ± 9 min. Medial pain relief was seen in all 46 patients with a significant decrease in visual analog scale (VAS) score and improvement of knee and function subscores of the American Knee Society Score. On final follow-up, radiographs showed an increase in medial joint space and an “obvious” correction of alignment in the lower extremity was seen in eight patients.

Liu et al. [30] reported on 84 patients and 111 knees who had undergone PFO with a follow-up of 1 year. This is one of the largest series reported, in terms of numbers. The majority were women (94 knees) and the average age was 59 ± 8.8 years; 94 knees were of KL grade 3 or 4. Radiographic assessments included KL grade, HKA and condyle-plateau (CP) angle, joint-space width and settlement value. They concluded that the factors affecting postoperative clinical outcome after PFO were Knee Society Score (KSS) clinical score, CP angle and medial joint-space width. The factors that influenced functional outcome included age, VAS score and KSS, HKA angle and settlement value. As objective radiological evidence, HKA angle and settlement value could be used as an important basis for patient selection for PFO. They also found that the odds for functional satisfaction increased by 7% for every year of age increase.

Utomo et al. [33] studied the outcomes of PFO on 15 patients with grade-4 OA. The average age was 61 ± 8 years. The radiographic assessment included the grade of OA, tibio-femoral angle, joint-space ratio (ratio of medial space/lateral space). Outcomes were assessed using Knee injury and Osteoarthritis Outcome Score (KOOS – pain score, symptom score, activities of daily living (ADL), sports and quality of life (QOL) scores), short-form (SF)-12 and Oxford Knee Scores and the above radiological parameters. They reported improvement in all scores and radiological parameters. However, they did not perform statistical tests to assess the significance of their results.

Yang et al. [18] reported the results of PFO in 110 patients including 76 women. The average age was 59.2 years and the mean follow-up was 49.1 months (24–189 months). Outcomes assessed were radiographic parameters including femoro-tibial angle (FTA) and joint space. Secondary outcome measures were VAS score, age, sex, laterality, the severity of OA, and KSS. At final follow-up, there was significant decrease in FTA, lateral joint space and VAS score and increase in medial joint space and KSS. Four patients had numbness in the leg postoperatively (two CPN and two superficial peroneal nerve injuries) and all resolved between 3 and 10 months. Sixteen patients had weakness but all returned to normal by 4 weeks.

Zou et al. [34], in their prospective comparison study between 40 patients with PFO and 52 patients with HTO for unilateral varus knee OA, found significant decrease in operation time, peri-operative bleeding, time to full weight-bearing, pain VAS score, FTA and complications and significant increase in the Japanese Orthopaedic Association score in the PFO group. While the authors conclude that short-term and long-term surgical effects of PFO on varus knee OA are superior to those of HTO; they recommend HTO for severely varus knees.

### Prognostic factors for a better outcome after proximal fibular osteotomy

Liu et al. [30] in their study determined what factors affected the outcome after PFO. They concluded that patients with a near-normal HKA angle showed better outcomes in joint function, which might be because PFO could only partially correct the varus deformity of the tibial plateau. In patients with a higher HKA angle, indicating severe OA, where a femoral condyle deformity is also frequently seen, PFO was not effective. For a given varus deformity, patients with higher settlement value fared better after PFO. This could be explained by the fact that the higher the settlement value, the more significant the effect of lateral fibular support and the better the outcome of PFO. While the preoperative KSS was the only independent factor associated with clinical satisfaction of patients, HKA angle and settlement value were the radiological factors that were found to be independent factors associated with significant functional improvement. The authors recommended using HKA angle and settlement value for patient selection.

### Complications

Peroneal nerve palsy has been reported following this procedure due to the proximity of the nerve to the proximal fibula. Yang et al. [18] reported 1.8% CPN and 1.8% superficial peroneal nerve palsy which recovered completely between 3 and 10 months. They also reported that about 14.5% had a weakness which returned to normal within 4 weeks.

One study [32] looked at the risk of injury to the CPN for fibular osteotomies in association with HTO and found that the branching of the CPN and innervation of muscles occurred in the proximal 82 mm and the maximum risk of injury to the CPN occurs with procedures at the proximal 15 cm of the fibula (24% compared to 3% if performed in the distal half). They recommended osteotomies in the distal half of the fibula to avoid this complication. But for PFO to be effective, the level of osteotomy that was most effective was found to be between 6 and 10 cm from the proximal tip of the fibula. Osteotomies below this level fail to unload the lateral tibial plateau and, hence, care should be taken to avoid nerve injury.

Another recommendation is to make the approach posterior to the coronal plane as approaching the fibula anterior to the coronal plane increases the chances of nerve injury [32]. An observation was that nerve supply to extensor hallucis longus (EHL) alone was affected in some cases as there is a tendency for the nerve supply of this muscle to be from separate single or multiple small branches which originate proximally [32, 35]. It is found to be consistently given off at 9 cm below the head of fibula [31]. Involvement of the EHL alone has a better prognosis than with an associated sensory loss. The recovery is delayed if there is sensory loss associated with EHL weakness [31]. One reason for the increased risk of these nerves is the decreased mobility of the nerves as they are tethered to the periosteum of the tibia with connective tissue [36]. Care must be taken while retracting to avoid traction or direct injury to this nerve [37].

## Scope for future research

There are still some unanswered questions that need to be answered by future studies as this procedure evolves and gains acceptance. Double-blinded randomized controlled trials are needed to evaluate the efficacy of the procedure and to establish its place in the management algorithm of OA of the knee and the following queries need to be addressed:

1. To assess if there are differences in response to surgery between the genders and between the younger and the older patient?
2. The usefulness of the procedure in young patients with medial OA and those with post-traumatic varus deformity with medial OA
3. Are the indications the same as those for medial UKA and HTO?
4. What is the maximum varus angle up to which the procedure can be successful?
5. What is the longevity of survival of this procedure before the patient requires TKA/HTO/UKA?
6. How does the presence of concomitant lesions, such as meniscal tears and/or ligament insufficiency, impact the outcomes of PFO?
7. Do combination strategies, like PFO combined with cell-based regeneration strategies/arthroscopic intervention, improve outcomes further?

## Conclusions

PFO appears to be an attractive option for medial compartmental OA of the knee. The current literature is limited to small case series and reports good outcomes with pain including correcting the varus deformity in medial OA. Further studies are required to establish the place of PFO in the management algorithm of medial compartmental OA before it can be recommended for routine clinical use.

## Data Availability

Not applicable.
